# MicroRNA Expression Profile in Endometriosis and Endometriosis-Associated Ovarian Cancer—Systematic Review

**DOI:** 10.3390/cells15040374

**Published:** 2026-02-20

**Authors:** Maria Szubert, Iwona Gabriel, Aleksander Rycerz, Monika Golińska, Jacek R. Wilczyński

**Affiliations:** 1 Department of Surgical and Oncologic Gynecology, 1st Department of Gynecology and Obstetrics, Medical University of Lodz, 251 Pomorska Street, 92-213 Lodz, Poland; aleksander.rycerz@umed.lodz.pl (A.R.); jrwil@post.pl (J.R.W.); 2 Borahay’s Lab, Department of Gynecology and Obstetrics, Johs Hopkins Research Institute, Baltimore, MD 21205, USA; 3 Department of Gynecology, Obstetrics and Oncological Gynecology, Medical University of Silesia, 40-055 Katowice, Poland; lapgynig@gmail.com; 4 Department of Biostatistics and Translational Medicine, Medical University of Lodz, 92-213 Lodz, Poland; monika.golinska@umed.lodz.pl; 5 Cancer Research UK Cambridge Institute, University of Cambridge, Cambridge CB2 0RE, UK

**Keywords:** miRNA, endometriosis, endometriosis-associated ovarian cancer, NGS

## Abstract

**Highlights:**

**What are the main findings?**

- Out of 2387 screened manuscripts, 13 studies originated from diverse geographic regions and included both patients with endometriosis and ovarian cancer diagnosed simultaneously or consecutively
- No consistent miRNA up- or downregulation was observed across all studies

**What are the implications of the main findings?**

- Current results in the literature related to miRNA do not allow conclusions to be drawn on the disrupted pattern leading from endometriosis to endometriosis-associated ovarian cancer
- The use of Next-Generation Sequencing (NGS) could help overcome limitations related to the selection of different reference genes in miRNA studies and improve the accuracy of relative expression analyses.

**Abstract:**

Endometriosis-associated ovarian cancer comprises a special group of ovarian cancers that most probably originate from endometriosis foci. Several in vitro studies have shown that microRNA (miRNA) plays an important role in this carcinogenesis. Our goal was to establish if a distinct miRNA profile can be associated with endometriosis and endometriosis-associated ovarian cancer with their potential causal relationship, and whether such a profile could be used clinically to prognose carcinogenesis in endometriosis foci. We conducted a systematic search according to PRISMA guidelines, registered at PROSPERO (number CRD42021245606). The search encompassed whole Pubmed, Cochrane and Medline databases to 1 May 2025 and the search strategy included the following [MeSH] terms: ‘miRNAs’ or ‘microRNAs’ or ‘miR’ and ‘ovarian cancer’ and ‘endometriosis’. Our ultimate inclusion criterion was that studies must simultaneously evaluate miRNA expression in endometriosis, regardless of its form and stage, and in endometriosis-associated ovarian cancer (EAOC), as only data generated under identical experimental conditions and using the same controls are truly comparable. The quality of the data was assessed using The Newcastle-Ottawa scale (NOS) and ROBINS-I tool. Our final analysis included 13 studies, comprising 608 patients and over 1000 miRNA molecules. Among those only five manuscripts presented raw data for each miRNA studied. Although several authors declared high sensitivity and specificity for one or more miRNA in distinguishing between endometriosis and endometriosis-associated ovarian cancer, a meta-analysis could not be performed due to the high heterogeneity of the studied samples. We concluded that there is not enough publicly available raw data to establish a set of miRNAs capable of differentiating between the two diseases and of prognosing carcinogenesis. The greatest limitation lies in the use of various standardized reference gene sets, which makes it impossible to compare relative miRNA expression across studies. New data from the next generation sequencing (NGS) experiments would overcome issues related to reference and control genes.

## 1. Introduction

Endometriosis is a benign condition with symptoms connected to the location of endometriosis foci, i.e., on utero-sacral ligament, in the ovary, and on the peritoneum in the pouch of Douglas [1]. There are several classification systems used to assess endometriosis [2]. The simplest to use is the classification according to location of the foci: peritoneal endometriosis, ovarian endometrioma (endometrial ovarian cyst) and deep infiltrating endometriosis [3]. Regardless of the form, endometriosis can have a negative impact on quality of life [4]. Frozen pelvis, the phenomena of pelvic organs attached to each other by an inflammatory process caused by endometriosis, raised serious doubts for years on the subsequent carcinogenesis in such remodeled tissues. Endometriosis-associated ovarian cancer (EAOC) was first described in 1925, and subsequently many theories were proposed to establish the cause of the transformation [5]. Although EAOC is generally rare, significantly higher incidence of clear-cell and endometrioid ovarian cancer was found in women with histologically proven endometriosis in a large population-based Dutch study [6]. In recent decades, several molecular pathways were studied to establish differences between eutopic endometrium (inside uterine cavity) and endometriotic lesions, and to understand why certain lesions (especially ovarian lesions) are more prone to undergo a malignant transformation [7,8,9]. Progression from endometriosis into endometriosis-associated ovarian cancer is now supported by epidemiological studies and molecular data and it is believed that the *ARID1A* (*AT-rich interactive domain 1A*) gene mutations represent the basis for the transition step: atypical endometriosis or borderline ovarian tumor [10,11,12]. ARID1A is involved in controlling gene activity by changing chromatin remodeling [12]. However, the development of cancer from endometriosis is a slowly progressing process that is difficult to detect through clinical evidence. The risk of ovarian cancer is about 2.5 times higher in patients with endometriosis than in the general population but this number is probably underestimated because of the methodological limitations. Not all women suffering from endometriosis-associated ovarian cancer had previously received a laparoscopic diagnosis of endometriosis [13]. Furthermore, large epidemiological studies have demonstrated that the presence of endometriosis multiplies the risk of ovarian cancer, renal cancer, thyroid cancer, brain tumor, melanoma and breast cancer [14]. Endometriosis-associated ovarian cancer (EAOC) is especially noticeable and retrieved as endometrioid ovarian cancer (EOC) and clear cell subtype of ovarian tumor (CCOC—clear cell ovarian cancer)—both subtypes constitute about 70% of EAOC [15,16,17,18].

MicroRNAs (miRNAs), as key regulators of numerous molecular processes, appear to play a pivotal role in transformation from endometriosis through its atypical forms into ovarian cancer [12]. MiRNAs are single-stranded, non-coding small RNA molecules (approximately 20–40 nucleotides in length) that regulate gene expression by inhibiting mRNA translation or facilitating the cleavage of target messenger RNA (mRNA) [19]. Discovered in 1993, they changed the way the regulation of our genome is perceived. That is why their discovery was honored by Nobel Prize for Prof. V. Ambros. MicroRNAs control biological pathways involved in proliferation, apoptosis, migration, cell cycle control, differentiation and angiogenesis, processes that under aberrant functioning can lead to pathological status, including malignant transformation [20]. Their upregulation and downregulation contributes to the promotion of tumor growth [21]. The upregulation of miRNAs typically downregulates tumor suppressor genes (*PTEN*, *BRCA1*, *PDCD4*), activates proliferative pathways (PI3K/AKT, MYC), drives epithelial–mesenchymal transition and metastasis or suppresses apoptosis. Downregulation of tumor-suppressive miRNAs usually removes the inhibitory control on the oncogenic pathways [19,21,22,23].

Consequently, dysregulation of miRNA expression may contribute to complex pathological processes, including the development and progression of endometriosis and its malignant transformation. In this context, Ohlsson et al. and Laudanski et al. demonstrated that the eutopic endometrium of women with endometriosis exhibits a distinct miRNA expression profile compared with that of healthy controls [24,25]. These findings suggest that altered miRNA regulation may already be present at early stages of the disease [24,25,26].

Moreover, the miRNA-200 family plays a crucial role in the epithelial–mesenchymal transition (EMT), a key mechanism involved in tissue invasion and tumor progression. Dysregulation of miRNA-200 family members has also been observed in endometriosis-associated ovarian cancer (EAOC), as reported by Suryawanshi et al. [27], further supporting their role in malignant transformation.

Consistent with these observations, our group demonstrated significant differences in miRNA expression between various endometriotic tissues and ovarian cancers of different histopathological origins [28,29]. Together, these studies highlight the importance of miRNA profiling in understanding the molecular mechanisms underlying endometriosis and EAOC. The clinical relevance of miRNAs has recently been described by Bendifallah and colleagues, who developed a saliva-based microRNA diagnostic test for endometriosis. This approach represents the first clinical implementation of a miRNA signature for non-invasive disease detection [30].

However, a knowledge gap remains regarding whether miRNA can predict subsequent carcinogenesis in endometriosis patients. Therefore, we conducted a systematic review with the aim of performing a meta-analysis to summarize current knowledge on the role of miRNAs in the transition from endometriosis to endometriosis-associated ovarian cancer. We also attempted to evaluate their potential utility as diagnostic markers capable of discriminating between these two conditions and identifying patients with an increased risk of ovarian cancer based on endometriosis.

## 2. Materials and Methods

### 2.1. Search Strategy

We performed a systematic search according to the PRISMA guidelines [31]. The study was registered at PROSPERO with the number CRD42021245606 and followed registered inclusion and exclusion criteria. The search was conducted in MEDLINE (through https://pubmed.ncbi.nlm.nih.gov/), EMBASE (through https://ovidsp-1dc2-1ovid-1com-1kxl4notq0486.han.cib.umed.lodz.pl/ovid-b/ovidweb.cgi (accessed on 1 May 2025)), and the Cochrane Library. The search encompassed databases from their inception up to the 1 May 2025. We used a combination of controlled vocabulary (MeSH terms) and free-text terms. The final PubMed search string included:

(“miRNAs” OR “microRNAs” OR “miR”

AND (“ovarian cancer” OR “ovary cancer” OR “ovarian neoplasms”)

AND (“endometriosis”)*

Reference lists of eligible studies and relevant reviews were additionally screened to identify any studies missed by the initial search.

All retrieved records were assessed by two independent reviewers MS and IG and discrepancies were resolved in a discussion with a third reviewer—AR. Next, all retrieved records were imported into Rayyan (search app that is designed to help researchers conduct meta-analyses by making the selection process easier and organized) [32]. By running the selection process through the app, we confirmed the meticulousness of our search.

All searched manuscripts are shown in Supplementary File S1.

### 2.2. Study Inclusion/Exclusion Criteria

The PICOS (abbreviation from: Patient/Intervention/Comparison/Outcome/Study) principle was adapted as follows: Patients = the same experimental condition for endometriosis and EAOC, that means we included studies on patients with endometriosis compared to patients with endometriosis-associated ovarian cancer OR studies on patients with endometriosis and ovarian cancer diagnosed simultaneously or consecutively, Intervention = miRNA expression studied on excised endometriotic and neoplastic tissue or in blood, Comparison = between patients with endometriosis and ovarian cancer, Outcome = differential miRNA expression, and Study design = only studies that simultaneously examined endometriosis and ovarian cancer patients, since we were convinced that only experiment settings with the same control genes and in the same meticulous conditions would enable us to draw appropriate conclusions regarding common miRNA profile in these two entities.

Inclusion criteria were as follows:

(a) Searched terms: miRNA in endometriosis and miRNA in ovarian cancer, miRNA in endometriosis-associated ovarian cancer (EAOC); miRNA in ovarian neoplasms and endometriosis (strings provided above).
(b) Full-text original articles in English with available dataset or raw data on miRNA expression in both diseases.

We implemented following exclusion criteria:

(a) Only abstracts;
(b) Articles not in English;
(c) A lack of statistical methods described;
(d) Case reports, animal studies, in vitro only studies, review, meta-analyses, or editorial articles;
(e) The markers were not microRNAs.

If possible, we tried to contact the authors of studies that were published via e-mail and asked them to provide their data.

**Figure 1:**
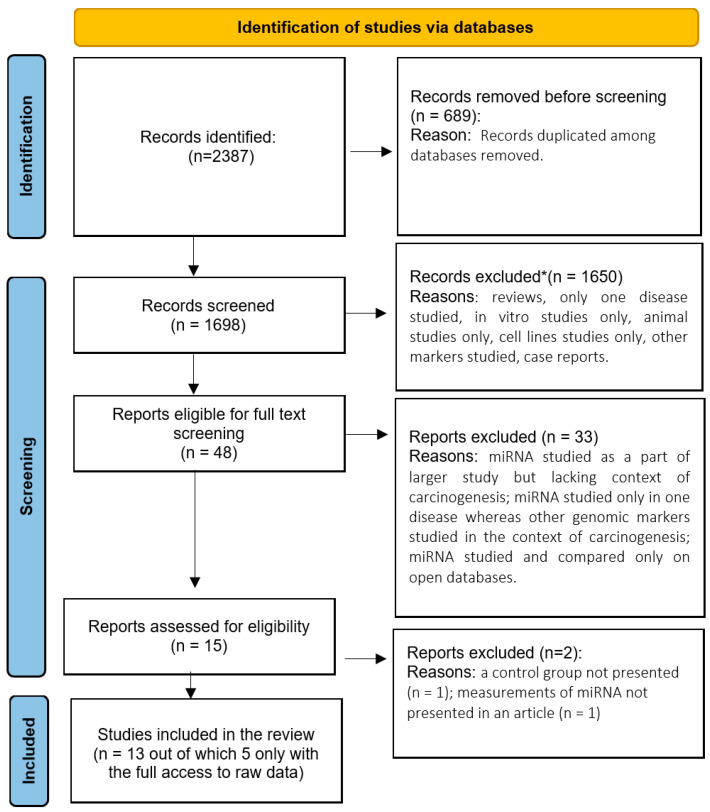
PRISMA flow diagram of searched databases (PRISMA 2020, according to Page at al. [33]). This work is licensed under CC BY 4.0. To view a copy of this license, visit https://creativecommons.org/licenses/by/4.0/ (accessed on 08/08/2025). *—screening performed manually and also through designed app Rayyan [32].

Detailed search strategy is described in Figure 1 (PRISMA flow diagram).

### 2.3. Data Extraction

All necessary information and data were extracted from the final eligible articles as follows: first author, year of publication, country of the study, number of cases and controls, characteristics of the patients and stage of the disease, research type, miRNA expression test methods, specimens, cut-off values, expression changes, and data needed for diagnostic meta-analyses (sensitivity and specificity). During the screening process, we mostly found review articles, which duplicated already known facts, the majority of them supported the role of miRNA in the malignant transition of endometriosis into ovarian cancer based on in vitro studies. In the final assessment we included 15 manuscripts, but two of them were excluded after full-text screening due to a lack of critical data. No randomized trials were identified within the selected studies (we assumed that we could find miRNA assessments within studies on new cancer protocols). All studies were observational cohort studies or cross-sectional studies, some of them with retrospective evaluations of endometriosis, some of them with the addition of an in vitro part, animal part or enrichment statistical analysis [27,28,34,35,36,37,38,39,40,41,42,43,44]—Table 1.

**Table 1:** The list of included miRNA studies.

Author, Year of Publication and Raw Data	Country	Number of Patients	miRNA Studied	miRNA Assessment Method	Sample Type	Cell Lines
Suryawanshi S. et al., 2013 [27]	USA	88	panel for 1113 miRNAs, then 23 detected by RT-qPCR	qPCR	human: plasma and FFPE, mice: whole blood	No
Wu RL. et al., 2015, [34] raw data: in GEO: GSE71477	USA	19	panel for 1156 miRNAs	qPCR	human: FFPE	No
Dong M. et al., 2015 [35]	China	36	miR-191	qPCR	human: serum, FFPE and commercial cell lines	HEK293T, CRL7566, CRL-11731
Tian X. et al., 2015 [36]	China	30	miR-191	qPCR	human: FFPE and commercial cell lines	HEK293T, CRL7566, CRL-11731
Braicu OL. et al., 2017 [37]	Romania	78	custom panel for 84 miRNAs	qPCR	human: FFPE	No
Hsu CY et al., 2018 [38]	Taiwan	9	miR-381, miR-203	qPCR	human: serum, stromal cells isolated from tissue and commercial cell lines	TOV21G, TOV112D
Nakamura N. et al., 2020 [39]	Japan	41	microarrays panel for 2578 miRNAs; miR-39-3p and miR-486-5p in qPCR	microarrays and qPCR	human: serum, peritoneal fluid and commercial cell line	EMOsis-CC/TERT
Kumari P. et al., 2021 [40]	India	40	miR-16, miR-20a, miR-99b, miR-125a, miR-143, miR-145	qPCR	human: fresh frozen tissue and FFPE	No
Szubert M. et al., 2023 [28] raw data published along the paper	Poland	135	array panel for 754 miRNAs; miR-1-3p, miR-125b-1-3p, miR-31-3p, miR-200b-3p, miR-502-5p, miR-503-5p and miR-548d-5p in qPCR	qPCR array and qPCR	human: FFPE	No
Takamizawa S. et al., 2023 [41]	Japan	64	array panel for 754 miRNAs; miR-146a-5p, miR-191-5p, miR-484 and miR-574-3p in qPCR	qPCR array and qPCR	human: serum	No
Collins KE. et al., 2023 [42] raw data in GEO: GSE230956	USA	35	Whole-miRNome sequencing (WMS)	RNAseq and qPCR	human: fresh frozen tissue and commercial cell lines	ES-2, TOV-21G, IGROV-1, SKOV3ip1, OVISE, OVAS, OVTOKO, KK, SMOV-2, A2780, A2780CR5, SKOV3, RMG-I
Talu ECK et al., 2025 [43] raw data published along the paper	Turkiye	33	miR-21 and miR-200b	qPCR	human: FFPE	No
Ravegnini G. et al., 2025 [44] raw data in GEO: GSE292134	Italy	37	Whole-miRNome sequencing (WMS)	Recover All™ total Nucleic Acid Isolation Kit (Thermo Fisher Scientific, Waltham, USA), run on NextSeq 500 high-output (Illumina, San Diego, USA)	human: FFPE	No

### 2.4. Trial Quality Assessment and Risk of Bias

We used Newcastle-Ottawa quality assessment scale (NOS) for cohort studies for the quality assessment of included papers (https://www.ncbi.nlm.nih.gov/books/NBK299087/ (accessed on 10 Aug 2025)). The Newcastle-Ottawa Scale quality instrument is scored by awarding a star for each positive answer. Possible total points are 4 stars for Selection, 2 points for Comparability, and 3 points for Outcomes. The total score range was from 0 (worst) to 9 (best) for case–control and cohort studies, with a score of at least 6 suggesting high quality (Table 2).

**Table 2:** The Newcastle-Ottawa quality assessment scale (NOS) for included manuscripts, according to Newcastle-Ottawa Scale Coding Manual For Cohort Studies. Available from: https://www.ncbi.nlm.nih.gov/books/NBK299087/ (accessed on 10 Aug 2025). Grading: Selection—possible max 4 stars; Comparability—possible max 2 stars, Outcome - possible max 3 stars. Lower scoring – when one or more numbered items in the NOS scale was not present in the assessed manuscript.

Author	Selection	Comparability	Outcome	Total
Suryawanshi S. et al., 2013 [27]	****	**	***	9
Wu RL. et al., 2015 [34]	**	**	**	6
Dong M. et al., 2015 [35]	**	**	**	6
Tian X. et al., 2015 [36]	***	*	*	5
Braicu OL. et al., 2017 [37]	****	**	***	9
Hsu CY. et al., 2018 [38]	**	*	*	4
Nakamura N. et al., 2020 [39]	***	**	**	7
Kumari P. et al., 2021 [40]	***	**	***	8
Szubert M. et al., 2023 [28]	***	**	***	8
Takamizawa S. et al., 2024 [41]	****	**	**	8
Collins KE. et al., 2023 [42]	**	**	***	7
Talu ECK. et al., 2025 [43]	**	**	***	7
Ravegnini G. et al., 2025 [44]	****	**	***	9

Eleven out of thirteen manuscripts achieved at least six points on the NOS. The remaining two scored lower because of methodological limitations. Specifically, Wu et al. included both HGOC and EAOC within a single study group and provided limited clinical characterization of the population [34], while Hsu et al. did not adequately report clinical characteristics of the studied cohort [38].

Next, we assessed the risk of bias using the ROBINS-I scale (Risk Of Bias In Non-randomized Studies of Interventions—a structured and standardized framework to assess case–control, cohort, and observational studies developed by Cochrane Bias Methods Group) [45,46]—Table 3.

Risk of bias according to the ROBINS-I indicated serious concerns in most studies, primarily due to methodological heterogeneity, insufficient reporting of raw Ct values [27,35,36,37,38,39,40,41] and small sample sizes [38,42]. We adopted and assessed each column as follows:

1. Confounding—confounding factors that can alter miRNA expression independently of the disease (patient’s related: age—described only partially in all manuscripts, data not comparable—mean vs. median, ethnicity—usually not given, menstrual and hormonal status—usually not described, stage of the disease; sample’s related: heterogeneity of the tissue, fresh/frozen/FFPE—usually properly described, storage conditions; laboratory related—different kits used—usually properly described, different methodology, lack of description of some steps).
2. Selection of participants—selection regarding: prospective [35,36,39,40,44] vs. retrospective [27,28,34,37,43] vs. not reported or cohort mixed [38,41,42], age matched vs. non-matched, randomly selected vs. consecutively, low number of participants.
3. Classification of exposure—whether the exposure was measured and categorized correctly (endogenous controls properly reported in only a few manuscripts, normalization, CT values, test used properly reported).
4. Deviations from the intended procedure—if there were major deviations from the planned protocol present.
5. Missing data—assessment if clinical data are present, measurement of outcomes—if all measurements and raw data provided, selection of reported results—if all results presented or only part of them (including Supplementary Files).
6. Overall bias—summarizing, overall judgment equals the highest level of bias in any domain.

Detailed presented and/or missing data are recorded in the Supplementary File Table S1 “Patients’ characteristic”.

Overall Risk of Bias was assessed as “serious” for all analyzed manuscripts as each scored “serious” in one or more domains. The assessment is presented in Table 3.

**Table 3:** Risk of bias assessed using ROBINS-I as follows: each domain is scored as: low risk of bias, moderate risk, serious risk, critical risk or no information. Low and moderate risk indicate confounders are controlled well.

Author	Confounding	Selection of Participants	Classification of Exposure	Deviations from Intended Exposure	Missing Data	Measurement of Outcomes	Selection of Reported Results	Overall Risk of Bias
Suryawanshi S. et al., 2013 [27]	moderate	moderate	moderate	low	serious	serious	serious	serious
Wu RL. et al., 2015 [34]	serious	moderate	moderate	low	serious	serious	serious	serious
Dong M. et al., 2015 [35]	serious	moderate	moderate	low	serious	serious	serious	serious
Tian X. et al., 2015 [36]	moderate	moderate	moderate	low	serious	serious	serious	serious
Braicu OL. et al., 2017 [37]	low	moderate	moderate	low	serious	serious	serious	serious
Hsu CY. et al., 2018 [38]	serious	moderate	moderate	low	serious	serious	serious	serious
Nakamura N. et al., 2020 [39]	moderate	moderate	moderate	low	serious	serious	serious	serious
Kumari P. et al., 2021 [40]	moderate	moderate	moderate	low	serious	serious	moderate	serious
Szubert M. et al., 2023 [28]	moderate	moderate	moderate	low	serious	serious	moderate	serious
Takamizawa S. et al., 2024 [41]	low	moderate	moderate	low	serious	serious	moderate	serious
Collins KE. et al., 2023 [42]	serious	moderate	moderate	low	serious	serious	moderate	serious
Talu ECK. et al., 2025 [43]	serious	moderate	moderate	low	serious	serious	moderate	serious
Ravegnini G. et al., 2025 [44]	low	moderate	low	low	serious	serious	moderate	serious

## 3. Results

Only 15 articles fulfilled the inclusion criteria. Among them one study included endometriosis for statistical comparisons only and did not assess its miRNA expression [47]. Another one did not present miRNA expression. Therefore, 13 manuscripts were included in the final analysis. In total, 608 patients with both entities studied in one study or both entities in one patient (coexisting endometriosis and EAOC) were studied as well as over 1000 miRNA subtypes. Relative expression and cut-offs for diagnostics models were only possible to obtain for five papers—among 13 manuscripts only five gave access to raw data.

MiRNAs were studied in three different approaches: one/two miRNA studied in the selected groups [35,36,38,43], miRNA set screened on the pooled groups and then the chosen miRNA studied in each patient [27,28,37], miRNA expressed in the Next Generation Sequencing (NGS) method in all patients (Colins et al. [42], Ravegnini et al. [44]).

We did not identify any miRNA whose upregulation or downregulation was consistently observed across studies. Comparisons between the groups were also impossible because different reference genes were used for normalization in the Quantitatively Real-Time Polymerase Chain Reaction (qPCR) assays. Proper normalization is essential to control for technical variability and ensure reliable and comparable gene expression results. It is worth mentioning that although all studies adequately described the normalization process, only consistent normalization using the same reference gene(s) allows reliable comparison of miRNA expression across experimental groups. Design of the analyzed experiments is presented in Table 1 (Section 2). The included studies applied heterogeneous approaches to patient and tissue selection. Biological material consisted of either fresh-frozen samples or postoperative formalin-fixed paraffin-embedded (FFPE) tissues; in several studies, commercially obtained control tissues were used [27,38]. Additionally, five studies assessed miRNA expression in peripheral blood, analyzing either serum or plasma samples (Table 4).

**Table 4:** miRNA expression in plasma or serum in the studied manuscripts.

Author	Number of miRNAs Studied	Control Gene Used	Changes in Endometriosis	Changes in Endometriosis Associated Ovarian Cancer (EAOC)
Suryawanshi S. et al., 2013 [27]	1113 in pooled screening cohort, then 23 detected by RT-qPCR	endogenous control miRNA, miR-132	upregulated top three: miR-16, miR-195, miR-191	upregulated top three: miR-21, miR-191, miR-16
Dong M et al., 2015 [35]	One only, miR191	RNU6B	upregulated	further upregulation
Hsu CY et al., 2018 [38]	Two—miR-203 and miR-381	RNU6	downregulated	downregulated
Nakamura N. et al., 2020 [39]	miR-92a-3p, miR-486-5p, miR-4484, miR-6821-5p, and miR-7108-5p	cel-miR-39-5p	endometriosis set as control tissue	miR-485-5p significantly upregulated
Takamizawa S. et al., 2023 [41]	Four miRNAs—miR-146a-5p, miR-191-5p, miR-484 and miR-574-3p	miR-16 (477860_mir)	none	upregulated: miR-146a-5p and miR-191-5p

Although each study demonstrated a clearly defined patient selection process, the absence of supplementary data (like age, BMI, stage of cancer) precluded meaningful clinical correlations. Detailed characteristics of the patients (median age, body mass index—BMI, FIGO stage—Fédération Internationale de Gynécologie et d’Obstétrique = International Federation of Gynecology and Obstetrics, or endometriosis stage) is presented in Table 1 in the Supplementary File, along with the full outcomes of the analyzed studies (Table S2 in Supplementary File).

As a control or comparison group, healthy ovarian tissue [27,28,43], healthy endometrium [40,43] or/and the serous type of high-grade ovarian cancer (HGOC) [27,28,36,37,38] was used. Unfortunately, sometimes we also found HGOC as a part of EAOC group [34,38,43]. Wu et al. did not present a control group, and high-grade ovarian cancer and endometriosis-associated ovarian cancer were mixed together as one group of ovarian cancer. In his work endometriosis coexisted with both histopathologically different ovarian cancers [34]. Collins et al. and Hirata et al. presented cases of endometriosis-associated ovarian cancer with simultaneously diagnosed endometriosis at the time of the same surgery, confirmed by pathological report during the final postsurgical assessment as studied group [42,47]. A summary of our findings regarding miRNA expression in tissue is presented in Table 5.

**Table 5:** miRNA expression in endometriosis tissue and endometriosis-associated ovarian cancer tissue.

Author	Number of miRNAs Studied	Control Gene Used	Changes in Endometriosis	Changes in Endometriosis Associated Ovarian Cancer (EAOC)
Suryawanshi S. et al., 2013 [27]	1113 in pooled screening cohort, then 23	endogenous control miRNA, miR-132	N/A	upregulated: miR-16, 21, 15b, and 195
Wu RL. et al., 2015 [34]	1156 in pooled screening cohort, then 7	miRNA in endometriosis samples; relative differential expression of the selected miRNAs in ovarian cancer was demonstrated by setting the expression level of endometriosis at 1.0.	expression levels set at 1.0	downregulated: miR-1, miR-133a, and miR-451, upregulated: miR-141, miR-200a, miR-200c, and miR-3613
Dong M. et al., 2015 [35]	One miRNA—miR-191	RNU6B	upregulated	further upregulation in comparison to endometriosis
Tian X. et al., 2015 [36]	One miRNA—miR-191	RNU6B	upregulated	further upregulation in comparison to endometriosis
Braicu OL. et al., 2017 [37]	84 in PCR array, 7 in qRT-PCR quantitative validation	the average Ct value of the cel-miR-39, SNORD68, SNORD95, SNORD96A and RNU6-6P	miR-93, miR-492	miR-93, miR-200c, miR-141, miR-492
Hsu CY. et al., 2018 [38]	Two miRNAs—miR-203 and miR-381	RNU6 (001093, Applied Biosystems)	miR-203 upregulated and miR-381 downregulated	miR-203 downregulated and miR-381 upregulated
Kumari P. et al., 2021 [40]	Six selected based on previous studies: miR-16, miR-20a, miR-99b, miR-125a, miR-143, and miR-145	U6 snRNA	downregulated: miR-16, miR-20a; upregulated: miR-145, miR-99b, miR-125a, and miR-143	downregulated: miR-16, miR-20a, upregulated: miR-99b, miR-125a, miR-145 and miR-143
Szubert M. et al., 2023 [28]	754 in screening cohort, then 7	hsa-miR-191-5p	downregulated: miR-125b-1-3p and miR-503-5p; upregulated: miR-200b-3p	downregulated: miRNA-200b and miRNA-31-3p, upregulated: miR-503-5p
Collins KE. et al., 2023 [42]	Small RNA sequencing (NGS)—over 43 million reads, log2 fold change > |1| identified 64 upregulated and 61 downregulated mature miRNA molecules	U6 snRNA	Endometriosis set as comparative tissue	top three upregulated: miR-10a-5p, miR-141-3p, miR-30a-5p, top three downregulated: miR-143-3p, miR-127-3p, let-7c-5p
Talu ECK. et al., 2025 [43]	Two miRNAs: miR-200b, miR-21	U6	upregulation of miR-200 b only when comparing endometriosis from endometriosis-only patients and endometriosis foci coexisting with CCOC	upregulated miRNA-21 and miRNA-200b but only in endometrium, not in cancer tissue
Ravegnini G. et al., 2025 [44]	global miRNA profiling using NGS method	statistically, using DESeq2 R-package (R version 4.4.0 (2024–04–24 ucrt) - "Puppy Cup")	13 deregulated miRNAs in endometriosis coexisting with cancer comparing to benign endometriosis: 9 were upregulated (miR-10a-5p, miR-126–5p, miR-141–3p, miR-144–3p, miR-144–5p, miR-183–5p, miR-200a-3p, miR-205–5p and miR-451a) and 4 downregulated (miR-345–5p, miR-361–3p, miR-483–3p, miR-675–3p)	upregulated in EAOC and in coexisting endometriosis tissue: miR-200a-3p, miR-141–3p, miR-183–5p, miR-10a-5p

The characteristics of the studied populations varied substantially. Lower Newcastle-Ottawa Score (NOS) scores and increased risk of bias according to the ROBINS-I tool were most commonly attributable to small sample sizes and insufficient reporting of key baseline data (age, BMI, stage of the disease, information about comorbidities, no clear exclusion criteria and lack of clear outcomes). These facts limited our ability to conduct the initially planned meta-analysis.

The included studies originated from diverse geographic regions, predominantly Asia and Europe, reflecting the higher prevalence of CCOC in East Asian populations. Most studies did not stratify miRNA expression data by ethnicity or geographic origin, which may influence miRNA profiles due to underlying genetic, epigenetic, and environmental differences. Therefore, the observed variability in miRNA expression across studies could partially result from population-specific factors.

The diagnosis of endometriosis was confirmed by laparoscopy or laparotomy. In contrast, details of ovarian cancer surgery were rarely reported, which limited the accuracy of FIGO classification; only 8 of the 13 studies provided information on the stage of ovarian cancer [27,28,34,35,39,41,42,44]. FIGO classification of ovarian cancer revealed an equal distribution of the disease among stages for endometriosis-associated ovarian cancer, but significant differences between endometriosis-associated ovarian cancer and high-grade ovarian cancer, where advanced stages were more dominant. An a ssessment of the stage of endometriosis was performed in only one study based on the American Society of Reproductive Medicine (ASRM) classification [35]; however, it was not reported which endometriosis tissue was used to study miRNA expression (endometrioma, peritoneal foci or deep infiltrating foci). Others reported studying ovarian endometrioma [28,42,43].

Since different concepts were adopted in nearly all studies, a quantitative comparison in a meta-analysis was not possible at the time of this review. Both control groups and control genes differed among studies (see Table 4 and Table 5 and Supplementary Table S2). Five studies reported miRNA expression in blood samples, and three of these also included tissue expression data [27,35,38]. Given the tissue-specific nature of miRNAs, cross-study comparisons were not feasible, despite all studies focusing on endometriosis and endometriosis-associated ovarian cancer. Some authors normalized miRNA expression to healthy ovarian tissue [27,28,37], others to endogenous endometrium [40], two of them set up endometriosis tissue as a “control” tissue and compared miRNA expression in endometriosis to expression in endometriosis-associated ovarian cancer or to expression in endometriosis foci localized close to endometriosis-associated ovarian cancer [34,42].

## 4. Discussion

### 4.1. Summary of Main Results

There has been ongoing debate regarding the potential risk of ovarian cancer development in patients with endometriosis and the methods by which clinicians can identify individuals with an increased susceptibility to carcinogenesis [48]. Thus, we sought to detect miRNAs engaged in the pathways leading from endometriosis into endometriosis-associated ovarian cancer. Our study is unique in its design because it brings together manuscripts that simultaneously focus on endometriosis and endometriosis-associated ovarian cancer, thereby creating comparable laboratory settings for both conditions, which is of utmost importance in miRNA studies. Since no single marker of endometriosis has been found to date, research efforts increasingly emphasize panels of markers, with special attention to miRNAs. Scientific development and falling prices of miRNA kits and NGS enabled several new studies to be conducted in the last few years. Since the last review published by Sheikhvatan M. et al. in 2020, data on miRNA in endometriosis and endometriosis-associated ovarian cancer has doubled [49]. According to Wu et al. identification of miRNAs that distinguish endometriosis-associated ovarian cancer from benign endometriosis may serve as potential biomarkers for ovarian cancer screening in patients with endometriosis. This fact might be especially important if we want to discuss the life-time follow-up of endometriosis patients [34].

Our systematic review demonstrated that a substantial knowledge gap remains, particularly regarding the identification of miRNA signatures that are shared or distinct between endometriosis and endometriosis-associated ovarian cancer. In addition, the overall quality of the reported data was influenced by a lack of clinical data and small groups of patients. Missing raw miRNA expression data were also one of the reasons why a meta-analysis on the role of miRNA in carcinogenesis pathway endometriosis-EAOC could not be conducted. Many approaches to the topic, although very interesting from a scientific point of view, underline the fact that we are still far from translating current knowledge about miRNA function in carcinogenesis into clinical practice.

### 4.2. Results in the Context of Published Literature

Nearly half of the analyzed papers presented in vitro or animal part of the study; researchers tried to focus on one or several miRNAs and its function in carcinogenesis [35,36,37,38,39,42]. This approach expands our knowledge on the cellular level. The role of miRNA is well established in different oncogenic pathways.

Tian et al. and Dong et al. analyzed miR-191 and concluded that this miRNA may play an important role in modulating the response of ovarian endometriosis and endometrioid carcinoma cells to death-inducing stimuli and that this effect occurs through the regulation of TIMP3 (tissue inhibitor of metalloproteinase-3, protein that influence extracellular matrix and tissue remodeling) [35,36]. Kumari et al. proved that miR-16 and miR-20a target many genes such as *HIF1A* (responsible for the adaptation of cells to hypoxia conditions), *COX2* and *TNF* (inflammation pathways), and *VEGF* (angiogenesis) [40]. miR-99b, miR-125a, miR-143, and miR-145 were reported to act on cell proliferation, tumor suppression, and tissue remodeling pathways [40]. Hirata et al. studied the influence of miR-21 on *PTEN* function and concluded that its mRNA and protein expression were increased by miR-21 knockdown in clear cell cancer cells [47]. *PTEN*—a well-known gene from oncological studies—is one of the most frequently mutated tumor suppressors in human cancers. It acts within the PI3K/Akt/mTOR pathway to control cell cycle progression, survival, and metabolism, preventing uncontrolled cell growth and promoting apoptosis (programmed cell death) [50,51]. It is also well studied in ovarian cancer [52] and its role was also confirmed by Braicu et al. and Takamizawa et al. and by our team in the bioinformatics sections of the papers [28,37,41]. Bioinformatics analyses using several free available databases like miRbase or miRNA Enrichment Analysis and Annotation Tool (miEAA) were conducted to find the most reliable targets of the studied miRNAs. Not surprisingly almost all miRNA targets have already been described as involved in carcinogenesis across different cancer types. MicroRNAs can influence key oncogenic pathways by regulating the mRNA expression of genes involved in transcriptional control, apoptosis, cell death, cell proliferation, and angiogenesis. Gaining further insight into their specific roles in endometriosis-associated ovarian cancer may therefore contribute to a better understanding of disease mechanisms [50,53,54].

### 4.3. Strengths and Weaknesses

Conducting a meta-analysis would require that a given miRNA be reported repeatedly using the same normalization strategy [55]. However, this criterion was not met. Therefore, the data were analyzed and presented only as a systematic review. The above-mentioned in vitro and animal parts of the study are interesting from a pathological point of view, but the methods of their description often raise concerns. In two manuscripts we found misleading data regarding how many patients and samples were used in which part of the manuscript (see NOS table). One study, which we excluded from further analysis, reported examining miRNA profiles in CCOC and endometriosis; however, endometriosis was included only as a comorbidity in the statistical analysis, and no endometriosis tissue was actually assessed for miRNA expression [47]. The manuscripts reporting cell-lines and in vitro studies as accompanying tissue miRNA analysis, tended to neglect clinical assessment of the involved patients (see Supplementary Table S1). This lack severely impacted drawing further conclusions. Although several miR databases link miRNA to diseases (miR2Trait, HMDD v.4.0 [56,57]), we suggest that all datasets should be deposited in an appropriate repository of the raw miR. This would significantly aid the scientific community in moving forward in this area of research. Being able to integrate data obtained by others would strengthen future publications.

As a recommendation for future research, it is worth emphasizing that responsible scientists have an obligation to publish raw data in formats that facilitate subsequent meta-analyses.

Many of the studies included do not report key parameters (age, BMI, FIGO stage, histotype), limiting the interpretability and comparability of their findings.

The use of different methods of sample analysis impacted downstream data interpretation. Consistent with the review by Vanhie et al. on miRNA in endometriosis, our analysis identified studies employing microarrays, RT-qPCR, and NGS, further complicating direct comparisons between datasets [58]. Researchers, like Talu et al. are also aware of the limitations coming from small groups or high costs of the miRNA expression studies [43]. That is why the majority of manuscripts reviewed by us adopted the pre-screening of pooled samples of 10–20 patients as a first step of the experiment, and only after confirming which miRNAs are up- or downregulated—at least 2-fold—was the final experiment on several chosen miRNAs conducted [27,28,34,37].

Several systematic reviews have already been published focusing on miRNAs in endometriosis; however, only one review exists for endometriosis and EAOC [59,60,61]. MicroRNAs have already been studied in nearly all tissues, even in saliva [62]. FFPE samples are used, because miRNAs are proven to be exceptionally stable after deparaffinization and can be readily and reliably detected in most tissues [63]. Three of the reported studies compared miRNA in blood and in FFPE samples. Data published by Surwayanshi et al. suggested however, that plasma and tissue samples had distinct miRNA expression profiles. They concluded that differentially expressed miRNA levels identified in FFPE samples cannot be simply extrapolated to plasma/serum samples [27]. Another controversial topic is endogenous control. All comparative miRNA studies require reactions to be performed with a control gene. Rnu6, which is used most often, cannot serve as a reliable normalization control according to Kaija et al. because of its changes in hypothermic and ischemic conditions [64]. miR-191 is also frequently used in miRNA studies [65], but it may not be suitable for endometriosis and EAOC. Its stability was well proven in bone marrow mesenchymal stromal cells [43], but on the other hand its influence on metalloproteinase-3 pathway may limit its use in studies on carcinogenesis [35]. There is also no consensus on housekeeping miRNAs used for plasma miRNA RT-qPCR data normalization [27]. Currently only the NGS technique enables data-based normalization (during statistical analysis of data) and physical normalization of DNA libraries, as presented by Collins et al. [42]. If NGS is not used, running qRT-PCR requires several obligatory steps to ensure proper normalization: identifying potential reference genes from the literature, validating them experimentally and utilizing multiple normalizers [66].

### 4.4. Implication for Practice and Future Research

After conducting the systematic search, we observed that the most relevant datasets investigating the potential involvement of miRNAs in carcinogenesis and endometriosis have been published within the last two years. Given that data from miRNA studies are expected to grow substantially in the coming years, future efforts should focus on identifying differences in miRNA expression among healthy ovarian tissue, benign endometriomas, and endometriosis foci coexisting with endometriosis-associated ovarian cancer. Ideally, these studies should employ next-generation sequencing approaches. More NGS publicly shared data is needed to improve miRNA’s accuracy in the prediction of carcinogenesis and to enable meta-analyses and further statistical evaluations. Such results may be implemented quickly into clinical settings, for example, FFPE endometriosis tissue (ovarian endometrioma mostly, as the tissue with the highest predisposition to molecular transformation into cancer [67]) can be tested for particular miRNAs expressions. An algorithm based on miRNA expression could help predict which endometriosis patient should undergo close surveillance when of childbearing age or which patients should be considered for a bilateral salpingo-oophorectomy as a prophylaxis, when in the perimenopausal/menopausal period. Since pathogenesis of each cancer is complex, miRNA solely is not an answer—each study should present as much clinical data as possible.

**Figure 2:**
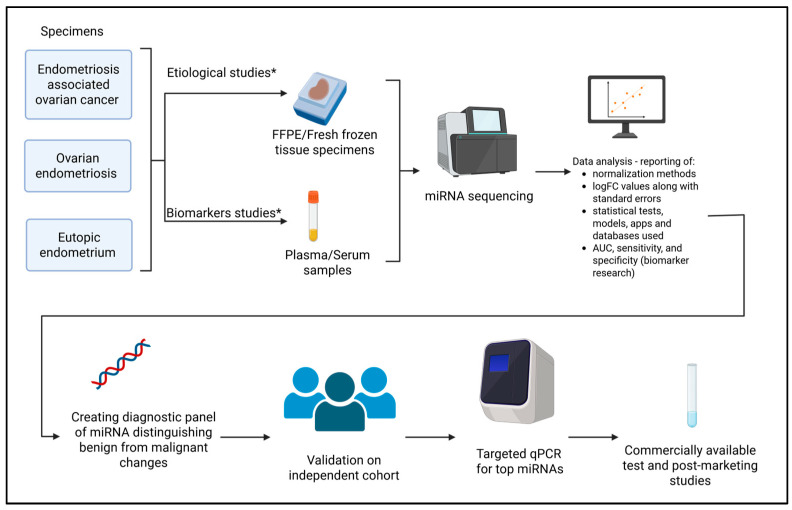
The proposed protocol for the development of miRNA-based diagnostic algorithm. * Data required: age, race, BMI, hormonal status, menstrual status, comorbidities (other cancers excluded), prospective study, and stage of the disease. An additional independent test set comprising 25–30% of the total cohort is recommended to validate the classifier and avoid overfitting. Created in Biorender. Rycerz Aleksander. (2025) https://BioRender.com/.

In Figure 2 we proposed the “ideal” pathway for developing an miRNA-based test discriminating endometriosis and EAOC and assessing the risk of carcinogenesis. It should be emphasized that the proposed diagnostic pathway is conceptual and hypothesis-generating in nature. Given the heterogeneity and limited clinical annotation of the currently available data, this framework is not intended for immediate clinical application but rather to guide future research and the design of well-controlled validation studies.

Examples of well-designed studies employing advanced miRNA-based diagnostic approaches include the recently developed saliva assay detecting an endometriosis-specific miRNA signature, as well as emerging research in breast cancer applying comprehensive miRNA profiling for diagnostic purposes. In particular, the study by Smyczynska et al. in BRCA-positive breast cancer patients illustrates the advanced stage of methodological development and clinical integration of miRNA research in breast cancer [68]. Comparable data for EAOC, as we presented, are poor. Collectively, such miRNA-driven diagnostic platforms, as already implemented in breast cancer, illustrate the growing integration of personalized medicine into clinical practice [59,69]. Future research should also include multi-ethnic cohorts or stratify analyses by ancestry to clarify the role of ethnicity and geography in miRNA dysregulation in EAOC. As this field is still in its infancy, we hope that this systematic review will stimulate further interest in the malignant transformation of endometriotic lesions. We also anticipate that data from rapidly developing spatial transcriptomics approaches will provide additional insight into this process [42,70]. Integrating information from different molecular levels—such as miRNAs and their target mRNAs—will help close the interpretative loop and improve mechanistic understanding.

Previous studies have also extensively explored the role of miRNAs in epithelial ovarian cancer, demonstrating their involvement in tumor progression, prognosis, and therapeutic response [71,72]. While some of these miRNAs overlap with those identified in endometriosis-associated ovarian cancer, the underlying biological context and clinical implications may differ substantially. Integrating insights from epithelial ovarian cancer research therefore provides valuable context, while also underscoring the need for disease-specific investigations focused on the unique pathogenesis of EAOC.

## 5. Conclusions

According to our systematic review, the amount of publicly available data is insufficient to define a set of miRNAs capable of distinguishing endometriosis from EAOC or predicting which endometriotic lesions are prone to malignant transformation. Although miRNAs have clear diagnostic and therapeutic potential [73,74], NGS-based studies on the molecular evolution of endometriotic tissue are still needed to provide meaningful clinical guidance.

Therefore, based on the quality assessment of the reviewed manuscripts, we propose a pathway for developing a clinically useful miRNA-based diagnostic panel:

1. Collection of FFPE samples from diseases of interest (endometrioma and EAOC) together with associated clinical data;
2. MiRNA next-generation sequencing (NGS) with appropriate statistical normalization;
3. Development of a diagnostic model estimating the probability of carcinogenic transformation;
4. Validating the test on an independent cohort.

This approach will enable a robust evaluation of the diagnostic potential of miRNAs in transition from endometriosis to ovarian cancer.

## Data Availability

No new data were created or analyzed in this study.

## Supplementary Materials

The following supporting information can be downloaded at: https://www.mdpi.com/article/10.3390/cells15040374/s1, File S1: Literature search. Excel: Tabel S1 Patients Characteristics, Table S2. Main outcomes of the studies—full version.

## Abbreviations

The following abbreviations are used in this manuscript:

Healthy—Healthy, tissue not specified, OH—healthy ovarian tissue, OE—ovarian endometriosis, EAOC—endometriosis-associated ovarian cancer, EOC—endometroid ovarian cancer, CCOC—clear-cell ovarian cancer, SOC—serous ovarian cancer, HGSOC—high-grade serous ovarian cancer, mixOC—mixed ovarian cancer, EH—eutopic endometrium from healthy patients, FFPE—formalin fixed paraffin embedded blocks/tissue, NOS—Newcastle-Ottawa scale.
